# 囊性纤维化跨膜转导调节子（CFTR）对肺癌A549细胞恶性特性的影响研究

**DOI:** 10.3779/j.issn.1009-3419.2018.02.03

**Published:** 2018-02-20

**Authors:** 惠 李, 颖 王, 佳丽 杨, 晓明 刘, 娟 石

**Affiliations:** 1 750004 银川, 宁夏医科大学临床医学院 College of Clinical Medicine, Ningxia Medical University, Yinchuan 750004, China; 2 750004 银川, 宁夏医学大学总医院 General Hospital of Ningxia Medical University, Yinchuan 750004, China

**Keywords:** 囊性纤维化跨膜转导调节子, 肺腺癌, A549细胞, Cystic fibrosis transmembrane conductance regulators, Lung neoplasms, A549 cells

## Abstract

**背景与目的:**

肺癌发病率逐年上升，有必要寻找新型的治疗靶点，而最新研究发现囊状纤维化跨膜转导调节子（cystic fibrosis transmembrane conductance regulator, CFTR）与多种肿瘤的发生和恶性转化有关。本研究探讨CFTR对肺癌A549细胞恶性特性的影响。

**方法:**

应用CCK8细胞增殖实验、细胞划痕实验、Transwell细胞侵袭实验以及克隆形成实验等方法分别检测CFTR的表达对非小细胞肺癌A549细胞的增殖、迁移、侵袭等细胞恶性特性的影响。同时通过免疫印迹（Western blot）分析*CFTR*基因表达对肿瘤干细胞相关转录因子表达的影响。

**结果:**

过表达*CFTR*基因显著抑制A549细胞的增殖、迁移、侵袭和克隆形成等肿瘤恶性特征，而RNA干扰A549细胞CFTR的表达导致细胞上述特征的明显增强。免疫印迹实验进一步发现*CFTR*基因过表达抑制A549细胞中干细胞相关转录因子SOX2和OCT3/4，以及细胞表面CD133蛋白的表达；相反，RNA干扰A549细胞中*CFTR*基因的表达增加SOX2、OCT4和CD133的表达。然而，免疫印迹和流式细胞术发现*CFTR*基因表达对A549细胞肺癌干细胞标志乙醛脱氢酶1的表达和阳性细胞数量无显著影响。

**结论:**

*CFTR*基因在肺癌A549细胞中具有抑制细胞恶性特征的作用，提示其可能是肺腺癌治疗的一个新的靶点，但其对其他肺腺癌细胞的作用与分子机制还有待进一步研究。

## Abstract (en)

**Background and objective:**

The incidence of lung cancer is gradually increased, and the cystic fibrosis transmembrane conductance regulator (CFTR) has recently demonstrated to have an implication in the deoncogenesis and malignant transformation of many types of cancers. The aim of this study is to investigate impacts of CFTR on the malignant features of lung adenocarcinoma A549 cells.

**Methods:**

The capacity of cell proliferation, migration, invasion and clonogenicity of non-small cell lung cancer A549 cells were detected by CCK8 cell proliferation assay, cell scratch assay, Transwell cell invasion assay and clone formation assay, respectively. Meanwhile, the effect of *CFTR* gene on the expression of cancer stem cell related transcriptional factors was also detected by immunoblotting (Western blot) assay.

**Results:**

An overexpression of *CFTR* gene in A549 cells significantly inhibited the malignant capacity of A549 cells, including potencies of cell proliferation, migration, invasion and colony formation; while knockdown of *CFTR* gene expression by RNA interference in A549 cells resulted in an opposite effect seen in above cells overexpressing *CFTR* gene. Mechanistically, immunoblotting assay further revealed that the ectopic expression of *CFTR* gene led an inhibitory expression of stem cell-related transcriptional factors SOX2 and OCT3/4, and cancer stem cell surface marker CD133 in A549 cells, while a knockdown of CFTR expression yielded a moderately increased expression of these gene. However, an alteration of *CFTR* gene expression had neither effect on the expression of putative lung cancer stem cell marker aldehyde dehydrogenase1 (ALDH1), nor the frequency of ALDH1A-positive cells in A549 cells, as ascertained by the immunoblotting assay and cytometry analysis, respectively.

**Conclusion:**

The CFTR exhibited an inhibitory role in the malignancy of lung adenocarcinoma A549 cells, suggesting that it may be a novel potential target for lung cancer treatment. However, its functions in other lung adenocarcinoma cell lines and its underlying molecular mechanisms require further investigation.

据世界卫生组织报道，在所有肿瘤中肺癌的发病率和病死率逐年上升，目前已居肿瘤病死率的首位^[1]^。同样我国近年来肺癌的发病率较几十年前已经增加了1.5倍，为增长最快的恶性肿瘤。基于靶向特异信号通路和免疫检查点的患者个体肿瘤精准靶向治疗因毒副作用小和患者受益率高而广受关注，但分子靶标的筛选是肿瘤的精准靶向治疗的基础。因此，寻找靶向肺癌治疗的有效潜在靶点对于肺癌防治的研究具有重要的意义^[2]^。

肿瘤的恶性转移和耐药性是抗肿瘤治疗失败的主要原因，而肿瘤组织中一群具有自我更新和分化能力的细胞亚群，即肿瘤干细胞（cancer stem cell, CSC）被认为在肿瘤的发生、发展、转移、复发等恶性变异和抗肿瘤治疗中发挥关键作用^[3]^。在过去的几年中越来越多的证据表明肿瘤干细胞在包括乳腺癌^[4]^、前列腺癌^[5]^、胰腺癌^[6]^和肺癌^[7]^中都发现有CSC的存在。同样肺癌干细胞在肺癌的恶性变化和抵抗放疗、化疗和靶向治疗的发展过程中发挥重要重要^[8]^。与其他类型肿瘤一样，目前对肺癌干细胞尚无特异性的标记物鉴定，尽管一些分子表面标志和干细胞相关转录因子的表达，如CD133、CD44、SOX2、OCT3/4和乙醛脱氢酶1（aldehyde dehydrogenase 1, ALDH1）的表达被认为是肺癌干细胞的潜在标志^[9]^，但它们的特异性有待进一步验证。

囊状纤维化跨膜转导调节子（cystic fibrosis transmembrane conductance regulator, CFTR）是一种氯离子通道蛋白，负责把氯离子从胞内运输到细胞外。CFTR蛋白还能调节其他通道的功能，例如在细胞膜上运输阳性离子钠离子。这些离子通道对于维系肺脏和其他器官上皮细胞的功能及组织稳态是必须的。已有研究表明*CFTR*基因突变导致的功能缺陷或缺失是引起肺脏囊状纤维化的原因。除此以外，越来越多的研究表明*CFTR*基因与许多肿瘤发生密切相关，在不同的肿瘤发生中起着促进或抑制肿瘤发生的作用^[10]^。新近研究证实CFTR是人类肠癌的抑癌基因^[11]^。抑制CFTR表达促进乳腺癌细胞的上皮-间质转化（epithelial-mesenchymal transition, EMT）特征^[12]^；而过表达CFTR抑制子宫内膜肿瘤细胞的增殖和迁移能力^[13, 14]^。这些结果表明CFTR在上述肿瘤中具有抑癌基因的功能。但是在前列腺癌和鼻咽癌（nasopharyngeal carcinoma, NPC）中，*CFTR*基因呈现原癌基因的功能，抑制*CFTR*基因表达可以通过抑制自噬而增强前列腺癌细胞对顺铂的敏感性^[15]^；而过表达CFTR增强鼻咽癌细胞的的迁移和侵袭能力^[16]^。上述结果清楚表明*CFTR*基因表达与肿瘤的恶性改变及耐药性密切相关，但在不同类型的肿瘤中可能发挥不同的作用功能与机制，即在不同的肿瘤中CFTR既可发挥着抑癌基因的作用也可能发挥原癌基因的功能。

在肺癌中，早期遗传相关性和基因多态性研究发现*CFTR*基因突变与肺癌发生风险有关，*CFTR*基因缺失突变的携带者患肺癌的风险较*CFTR*基因正常的人群更低^[17]^。但是随后的表观遗传学研究发现非小细胞肺癌（non-small cell lung cancer, NSCLC）患者*CFTR*基因启动子的甲基化水平显著高于健康人群，而启动子的甲基化抑制靶基因的表达，因此这种启动子甲基化导致的CFTR表达下降可能与肺癌的发生有关^[18]^。这些研究结果明确*CFTR*基因与肺癌的发生密切相关，但其在肺癌发生发展中的作用目前还未有深入的研究。因此本研究以肺腺癌细胞系A549细胞为研究对象，通过Western blot法、CCK8实验、划痕实验、克隆实验、Transwell实验和流式细胞术等方法探讨*CFTR*基因对肺癌细胞恶性及干细胞特性的影响。

## 1 材料与方法

### 1.1 材料

#### 1.1.1 细胞系、腺病毒与抗体

肺癌细胞系A549（CCL#185）购自American Type Culture Collection（ATCC, Mannasas, VA, USA）。腺病毒空载体Ad/BgLII、Ad/CFTR过表达腺病毒载体CFTR和CFTR表达干扰腺病毒载体Ad/CFTRi均实验室自行构建^[19]^。Ad/BgLII为E3区缺失的人源5型腺病毒空载体对照；Ad/CFTR为E3区缺失的CMV启动子驱使CFTR cDNA（序列号M28668.1，GI:180331）的过表达载体；Ad/CFTRi为E3区缺失的小鼠U6启动子驱使的靶向人CFTR mRNA的5’GGAAGAATTCTATTCTCAATCCAAT3’序列的短发卡RNA（shRNA）。实验中所用抗体见表 1。

**1:** 实验中使用抗体信息表 Information of antibodies used in present study

Antigen	Host	Vendor	Catalog No	MW (kDa)
ALDH1A	Rabbit	Boster	BA3672	55
CD133	Rabbit	Abcam	Ab19898	110
CFTR	Mouse	Millipore	M3A705583	170
OCT3/4	Rat	RD Systems	MAB1759	46
SOX2	Rabbit	Proteintech	11064-1-AP	34
Beta-actin	Rabbit	Proteintech	20536	42

#### 1.1.2 试剂

实验中所用的试剂有RPMI 1640基础培养基（Glbco），胎牛血清（FBS）（Hyclone），Western Lighting ECL PLUS等；所用试剂盒为全蛋白提取试剂盒、BCA蛋白定量试剂盒（凯基生物公司）。

### 1.2 方法

#### 1.2.1 A549细胞培养

将A549细胞系培养于含10%胎牛血清、100 IU/mL青霉素及100 IU/mL链霉素的RPMI-1640培养液的100 mm培养皿中，在5%CO_2_和95%空气的湿润环境的培养箱中培养。

#### 1.2.2 细胞增殖能力检测

细胞增殖能力实验利用全式金CCK8细胞增殖试剂盒测定。简单描述如下：将A549细胞用胰酶消化并接种在96孔板中，密度为每孔6×10^3^，每孔含100 µL含10%血清的RPMI-1640培养基。待细胞贴壁后分别加入100 µL含有相同浓度的腺病毒Ad/BgLII、Ad/CFTR、Ad/CFTRi（感染复数为100）。培养板边缘的孔利用无菌PBS填充，并且同时设空白对照（仅加含血清RPMI-1640培养基），继续培养细胞48 h，实验终止前每孔加入10 µL CCK8试剂后，37 ℃分别培养1 h后终止培养，并用blank对照孔调零，在酶标仪上450 nm测定各孔的吸光度（optical density, OD），然后用相对应的OD比值来表示细胞增殖能力大小，每组取各孔的平均值，然后比较各处理组增殖能力。

#### 1.2.3 克隆形成抑制实验

使用克隆形成实验来测定各处理组A549细胞的干细胞特性。100 mm平皿培养A549细胞，细胞密度为80%-90%时，每个平皿中分别加入感染复数为100的Ad/BgLII、Ad/CFTR、Ad/CFTRi腺病毒。48 h后用胰酶消化并接种于六孔板中，每孔密度为3×10^3^。连续培养细胞10 d，每三天用含血清RPMI-1640培养基给细胞换液一次。弃去培养基，细胞用PBS漂洗细胞两遍。然后用4%多聚甲醛在室温下固定5 min，去除固定液，然后将细胞用0.5%结晶紫染色并在室温下温育30 min，小心弃去染色液，用水漂洗细胞以去除染色液。最后将染色的六孔板置于空气干燥过夜，次日对各处理组的细胞拍照后，并对各处理组的细胞克隆团数进行计数。

#### 1.2.4 细胞划痕实验检测细胞迁移能力

将A549细胞于100 mm的平皿中培养，细胞密度为80%-90%时，用200 μL无菌枪头对A549细胞进行十字划痕，用预热的PBS洗涤3次除去未粘连的细胞，然后用含血清RPMI-1640培养液给细胞换液且每个平皿中分别加入感染复数为100的Ad/BgLII、Ad/CFTR、Ad/CFTRi腺病毒。用10倍放大倍数的显微镜分别于0 h和48 h拍照观察各处理组细胞迁移能力的差异。

#### 1.2.5 Transwell实验

使用Transwell迁移室进行侵袭测定，实验分别分为实验组（Ad/CFTR、Ad/CFTRi）和空白对照组（Ad/BgLII），每组设置3个复孔。Transwell上室用基质胶（BD Martrigel）与预冷的无血清RPMI-1640按1:8稀释，每孔中加入稀释好的基质胶60 μL，放37 ℃、含5%CO_2_的培养箱中孵育5 h后，吸出小室中残余液体，每孔再加入70 μL无血清RPMI-1640培养基，30 min后吸去培养基。各处理组处理细胞48 h后，用胰蛋白酶消化并制作细胞悬液，均加入100 μL（3×10^4^）的细胞悬液于Transwell小室（孔径8 μm）的上腔，加入600 μL含30%FBS的完全培养基于小室下腔，放置于37 ℃、含5%CO_2_的培养箱中培养12 h，用PBS洗涤后加入4%多聚甲醛固定20 min，0.5%结晶紫染色液染色10 min，然后用棉签轻轻擦去上室内未侵袭细胞，用10倍放大倍数的目镜在显微镜下拍照对各处理组细胞进行计数比较各组侵袭能力的差异。

#### 1.2.6 利用Western blot技术检测CFTR及肿瘤干细胞相关转录因子和细胞表面标志的表达

用腺病毒Ad/BgLII、Ad/CFTR、Ad/CFTRi处理A549细胞48 h，吸取培养基，用预冷的PBS清洗细胞3次，利用全蛋白裂解液在冰上裂解细胞，然后用细胞刮刮下裂解物。吸取细胞裂解物至预冷的EP管中，4 ℃，12, 000 *g*离心10 min，取上清BCA法测蛋白浓度，然后取上清与蛋白上样缓冲液5:1混匀，70 ℃水浴锅煮蛋白30 min。取等量的处理好的蛋白于SDS-PAGE聚丙烯酰胺凝胶电泳，然后将它们转移到PVDF膜上，2 h后将膜于5%脱脂牛奶封闭2 h，加入合适稀释的表 1的一抗孵育4 ℃过夜，次日室温平衡半小时，然后用PBST洗膜3次，每次10 min。加辣根过氧化物酶标记的相应二抗室温孵育2 h，PBST洗膜10 min/次，共3次；使用ImageJ Software 1.46版通过光学密度测定来定量蛋白质表达水平。灰度值变化计算为每个样品的净强度除以各自内部对照（β-肌动蛋白）之前的比例。以上所有实验均重复3次。

#### 1.2.7 ALDH1

流式细胞术分析ALDH1阳性细胞亚群ALDH1A流式细胞术用来分析A549细胞中可能肺癌干细胞亚群。用感染滴度为100的腺病毒Ad/BgLII、Ad/CFTR、Ad/CFTRi分别感染A549细胞后48 h，胰酶消化收集细胞后计数，按ALDEFLUOR™ (Stem Cell Technology，产品号#01711)试剂盒的说明书要求操作，利用BD流式细胞分析系统（CyFlow Cube 15）对染色后的细胞进行流式细胞术分析ALDH1阳性细胞的百分数。实验重复2次。

### 1.3 统计学方法

采用统计软件SPSS 19.0软件进行数据分析，计量资料采用均数±标准差（Mean±SD）表示，组间比较采用*t*检验，以*P* < 0.05为差异有统计学意义。

## 2 结果

### 2.1 CFTR对肺癌A549细胞增殖能力的影响

利用CCK8法检测CFTR对肺腺癌细胞株A549细胞增殖能力的影响。CCK8细胞增殖实验结果表明：与空白对照组Ad/BgLII感染细胞组相比，Ad/CFTR感染细胞的增殖能力显著下降，而Ad/CFTRi感染组的细胞增殖能力明显增加（*P* < 0.05）（图 1）。

**1:**
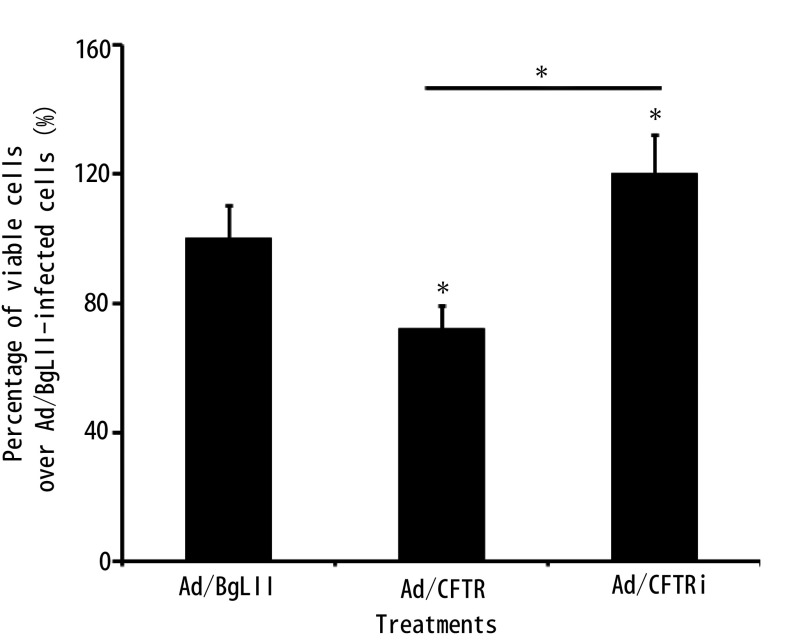
CCK8检测CFTR对肺腺癌增殖能力的影响。CCK8结果显示CFTR过表达明显抑制肺腺癌A549细胞增殖，而shRNA干扰下调CFTR表达促进A549细胞的增殖。*：表示组间比较，*P* < 0.05。 The effect of CFTR on the proliferation of A549 cells determined by a CCK8 assay. The results showed that the overexpression of CFTR significantly inhibited the proliferative ability of A549 cells, while knockdown CFTR by shRNA enhanced the proliferative capacity of A549 cells. Compared between groups, ^*^P < 0.05.

### 2.2 CFTR抑制肺腺癌A549细胞的迁移、克隆和侵袭能力

利用细胞的迁移、克隆和侵袭能力的改变分析CFTR对A549细胞恶性特性的影响。细胞划痕实验分析CFTR对A549细胞迁移能力的影响。与对照组Ad/BgLII感染细胞比较，Ad/CFTR过表达组处理A549细胞48 h后，细胞迁移能力受到明显降低；而Ad/CFTRi干扰组处理A549细胞后，细胞迁移能力增加（*P* < 0.05）（图 2A）。同样，用Transwell实验检测Ad/CFTR对A549细胞侵袭能力的影响，结果发现与对照组比较，Ad/CFTR过表达组处理A549细胞48 h后细胞侵袭能力明显下降；Ad/CFTR干扰组与对照相比细胞侵袭能力明显增强（*P* < 0.05）（图 2B）。细胞克隆实验进一步检测CFTR对A549细胞克隆能力的影响结果也表明Ad/CFTR过表达组与对照组相比，细胞克隆数显著减少，并且克隆体积明显变小；而Ad/CFTR干扰腺病毒载体处理A549细胞与对照组相比，细胞克隆数显著增多，并且克隆体积明显增大（图 3）。

**2:**
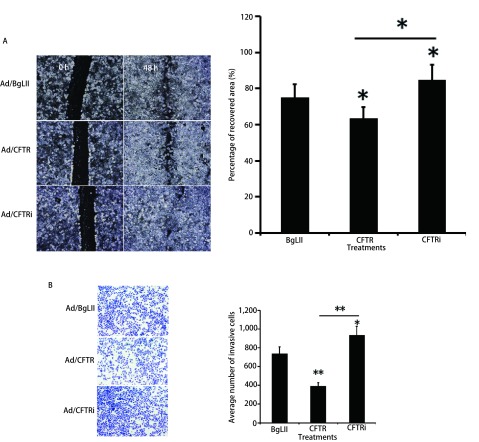
CFTR对肺腺癌A549细胞迁移和侵袭能力的影响。细胞划痕（A）和Transwell实验（B）表明CFTR抑制A549细胞的迁移（A）和侵袭（B）能力，而shRNA干扰下调CFTR表达促进A549细胞的迁移（A）和侵袭（B）能力。*：表示组间比较，*P* < 0.05；**：表示组间比较，*P* < 0.01。 Effects of CFTR on the capacity of migration and invasion in A549 cells. Cell scratch (A) assay and transwell assay (B) were used for determining the capacity of CFTR on cell migration (A) and invasion (B). The results demonstrated that an overexpression of CFTR significantly inhibited the capacity of migration (A) and invasion (B) in A549 cells, while knockdown CFTR by shRNA enhanced the ability of migration (A) and invasion (B) in A549 cells. Compared between groups, ^*^P < 0.05; ^**^P < 0.01.

**3:**
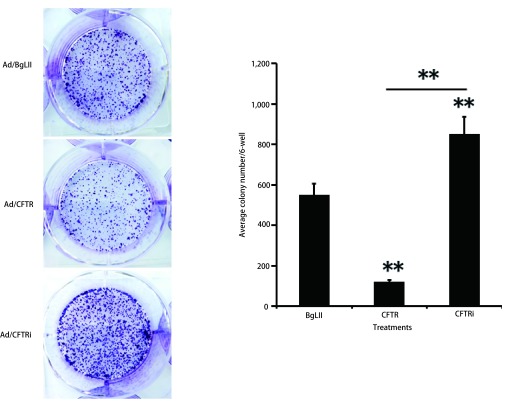
CFTR对肺腺癌A549细胞增殖能力的影响。细胞克隆形成实验显示过表达CFTR能够显著减低A549细胞克隆的形成，而shRNA干扰下调CFTR表达增强克隆形成能力。**：表示组间比较，*P* < 0.01。 Effects of CFTR on clone formation in A549 cells. Cell clonogenicity assay demonstrated that an overexpression of CFTR significantly inhibited the clone formation in A549 cells, while knockdown CFTR by shRNA increased clone formation in A549 cells. Compared between groups, ^**^P < 0.01.

### 2.3 CFTR抑制SOX2、OCT3/4等干细胞相关转录因子和CD133、ALDH1等干细胞标志基因的表达

SOX2和OCT3/4为干细胞主要相关的转录因子，而CD133和ALDH1是包括肺癌干细胞在内的肿瘤干细胞的主要分子标记，其表达与肺癌细胞的侵袭、克隆形成、耐药等肿瘤恶性特性密切相关。免疫印迹结果显示，当用腺病毒Ad/BgLII、Ad/CFTR、Ad/CFTRi分别感染A549细胞48 h后，与空白对照Ad/BgLII组相比，Ad/CFTR过表达组SOX2和OCT3/4等干细胞相关转录因子基因的表达，以及肺癌干细胞表面标志分子CD133的表达都有所下降，而Ad/CFTRi干扰组中上述蛋白的表达量有所增加（图 4）。值得注意的是与对照组相比Ad/CFTR过表达组与Ad/CFTRi干扰组，肺癌干细胞标志基因ALDH1的表达量均未发生明显变化（图 4）。流式细胞术分析进一步证实ALDH1的免疫印迹结果，即与Ad/BgLII对照组比较，Ad/CFTR和Ad/CFTRi感染组A549细胞的ALDH1阳性细胞亚群的百分率未有显著改变（图 5）。

**4:**
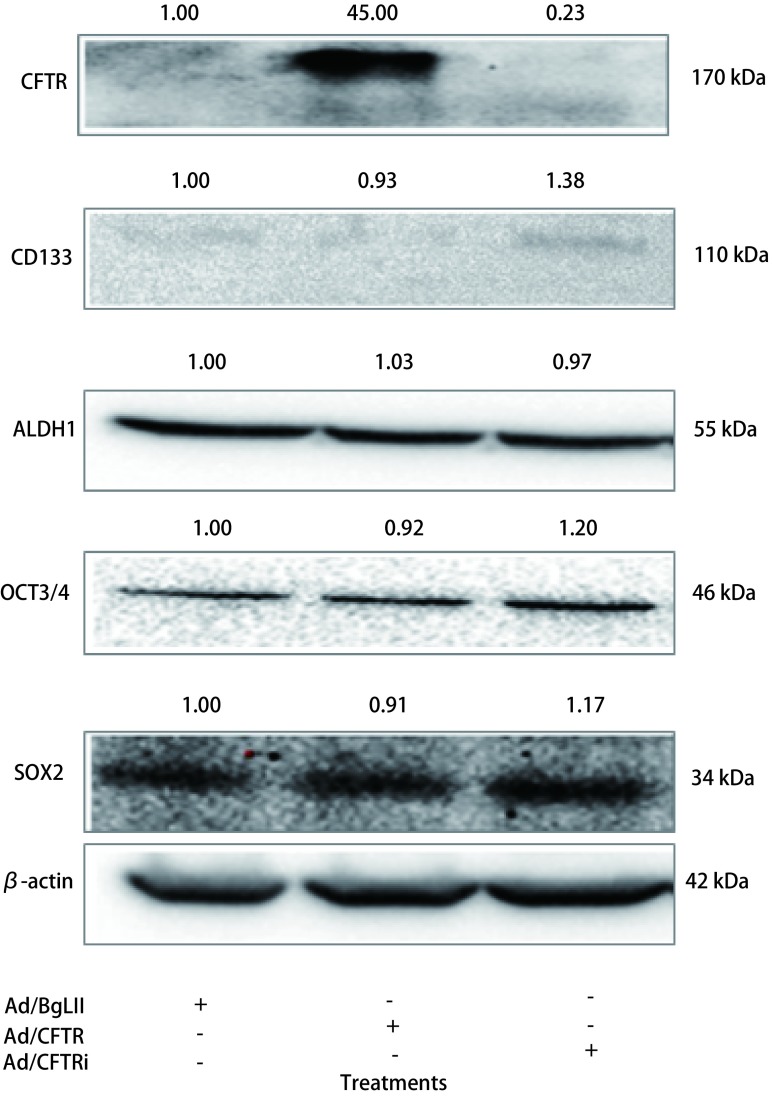
免疫印迹法检测肿瘤干细胞相关转录因子和肿瘤干细胞标记基因的表达。免疫印迹实验说明过表达CFTR抑制干细胞相关转录因子SOX2和OCT3/4，以及肿瘤干细胞标志CD133的表达，而shRNA干扰下调CFTR促进上述基因表达。但是CFTR对ALDH1的蛋白表达无影响。蛋白印迹上方数值为灰度分析后各种目的蛋白与Ad/BgLII感染细胞比较的相对表达变化倍数。 The expression of stem cell related transcriptional factor genes and CSC markers detected by an immunoblotting assay. The immunoblotting results showed that an overexpression of CFTR led a moderately decreased expression of SOX2, Oct4 and CD133 in A549 cells, while knockdown of CFTR by shRNA resulted in a slightly increased expression of above genes. However, the CFTR had no effect on the expression of ALDH1. The number on the top of each blot represented as a relative fold of change of the protein of interest over the cell infected with Ad/BgLII as determined by a densitometeric assay.

**5:**
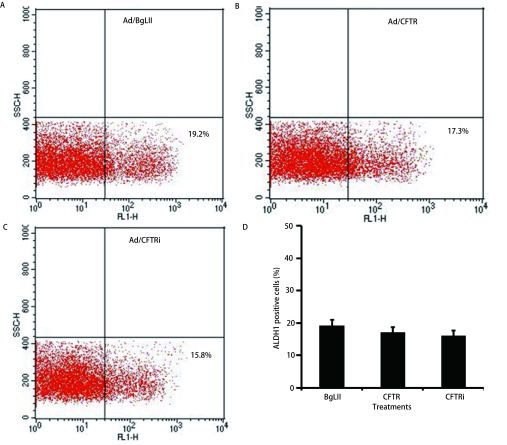
流式细胞术分析A549细胞的ALDH1阳性细胞率。流式细胞术分析Ad/BgLII（A）、Ad/CFTR（B）和Ad/CFTRi（C）病毒分别感染的A549细胞ALDH1细胞的阳性率。A-C图中左下角数值为ALDH1阳性细胞的百分率。D为各处理组ALDH阳性细胞的平均百分率。组间比较无统计学意义。 Percentage of ALDH1-positive cells in A549 cells determined by a cytometric assay. The cytometric analysis of Ad/BgLII (A), Ad/CFTR (B) and Ad/CFTRi (C)-infected A549 cells exhibited no significant difference in the ALDH1-positive cell fraction (D), suggesting the CFTR had no effect on the ALDH1 expression in A549 cells in this study.

## 3 讨论

肺癌是一种死亡率高的呼吸系统恶性肿瘤，近年来国内外的发病率都有所上升^[1]^。转移性肺癌及靶向治疗和化疗耐药性的发展，意味着肺癌目前仍无法治愈，患者预后不佳，5年生存率 < 20%^[20]^。实验和临床研究表明，肿瘤干细胞具有使其能够在许多常用的癌症治疗剂中存活的特性。肿瘤干细胞是肿瘤复发、转移等恶性转化和肿瘤细胞获得抗肿瘤治疗能力的根源。此外，肿瘤干细胞所具有的特性可以高度预测患者的总体存活率^[21]^。因而针对肿瘤恶性转化和干细胞的靶向治疗为癌症的治疗带来了希望。

本研究以肺腺癌细胞系A549细胞为研究对象，通过免疫印迹法、CCK8实验、划痕实验、克隆实验、Transwell和流式细胞术等实验等方法探讨CFTR对肺癌细胞恶性特性的影响。研究发现CFTR不仅能够改变肺腺癌肿瘤细胞恶性特性，还可以降低干细胞修改转录因子基因*SOX2*和*OCT3/4*，以及肺癌干细胞表面标志基因CD133的表达。结果显示CFTR能够显著抑制A549细胞的恶性转化能力。细胞划痕实验、Transwell实验和克隆形成实验分别证明CFTR能够降低A549细胞的迁移、侵袭和克隆形成能力，表明*CFTR*基因在肺腺癌A549细胞中发挥抑癌基因的作用，能够体外抑制肺癌A549细胞的恶性特性，可能是抑制肺腺癌细胞恶性转化的一个潜在的新型靶点。

CFTR是受cAMP调节的ATP门控氯离子通道蛋白，在1989年被克隆和鉴定。它的突变最初被认为可引起囊状纤维化疾病，随后研究发现CFTR失调与很多疾病有关，如COPD、肺纤维化以及肿瘤等^[22]^。最近研究发现*CFTR*基因在众多的肿瘤类型中发挥关键作用，CFTR已被确定为结直肠癌的候选驱动基因，有研究发现*CFTR*基因敲除小鼠与正常小鼠相比结肠中产生更多的肿瘤，表明CFTR是肠道肿瘤的抑制基因^[11]^。同样，CFTR在乳腺癌和子宫内膜瘤中都具有抑癌基因的作用^[12-14]^，而我们的研究结果CFTR在肺腺癌A549细胞中的作用与在肠道癌细胞、乳腺癌细胞和子宫内膜瘤细胞中的作用相一致。但是本研究只对*CFTR*基因在*KRAS*突变的A549细胞中的作用进行了探讨，CFTR是否在其他肺癌细胞具有同样的抑癌基因作用需要进一步的研究。

CSC理论认为肿瘤发生和维持是由于未分化的肿瘤干细胞群体的存在。由于细胞周期缓慢，增殖较低以及DNA修复和抗凋亡基因表达增加，CSC可能与治疗的复发、恶性转化和耐药性密切相关，其对放射化学疗法的细胞毒性具有天然抗性。到目前为止，还没有确定一个CSC的通用标记，尽管存在许多认定的CSC标记物，但是缺乏对NSCLC的精确标志物^[23]^。SOX2、OCT3/4等干细胞相关转录因子，CD133和ALDH1分子标志均被认为是肿瘤干细胞表面的重要标记，是CSC调控因子，可作为肿瘤扩散中的重要指标。已有研究表明SOX2、OCT3/4等干细胞相关转录因子在NSCLC细胞中高表达^[24]^；而CD133是肺癌干细胞的主要表面标志之一，在肺癌干细胞中高表达。这与本研究的结果相一致。但是我们的研究发现在A549细胞中ALDH1的表达未随CFTR介导的A549细胞恶性特征的改变而改变，这可能与ALDH存在多种亚型或不同肺癌细胞类型有关^[25]^。而ALDH1A只是众多ALDH亚型中的一种，在A549细胞中其表达与CFTR无关。总之，本研究发现*CFTR*基因的表达与肺腺癌A549细胞的恶性转化密切相关，CFTR在A549细胞中发现抑癌基因的作用，能够抑制A549细胞的恶性转化，但其深层次的分子作用机制还有待进一步探讨。
